# Neonatal mortality rate and determinants among births of mothers at extreme ages of reproductive life in low and middle income countries

**DOI:** 10.1038/s41598-024-61867-w

**Published:** 2024-06-01

**Authors:** Tadesse Tarik Tamir

**Affiliations:** Department of Pediatrics and Child Health Nursing, College of Medicine and Health Sciences, University of Gondar, Gondar, Ethiopia

**Keywords:** Neonatal mortality, Extreme age, Reproductive life, Low and middle income, Health occupations, Medical research

## Abstract

Neonatal mortality, which refers to the death of neonates during the first 28 completed days of life, is a critical global public health concern. The neonatal period is widely recognized as one of the most precarious phases in human life. Research has indicated that maternal extreme ages during reproductive years significantly impact neonatal survival, particularly in low- and middle-income countries. Consequently, this study aims to evaluate the neonatal mortality rate and determinants among neonates born to mothers at extreme reproductive ages within these countries. A secondary analysis of demographic and health surveys conducted between 2015 and 2022 in 43 low- and middle-income countries was performed. The study included a total sample of 151,685 live births. Researchers utilized a multilevel mixed-effects model to identify determinants of neonatal mortality. The measures of association were evaluated using the adjusted odds ratio within a 95% confidence interval. The neonatal mortality rate among neonates born to mothers at extreme ages of reproductive life in low- and middle-income countries was 28.96 neonatal deaths per 1000 live births (95% CI 28.13–29.82). Factors associated with higher rates of neonatal mortality include male gender, low and high birth weight, maternal education (no or low), home deliveries, multiple births, short preceding birth intervals, lack of postnatal checkups, and countries with high fertility and low literacy rates. This study sheds light on the neonatal mortality rates among neonates born to mothers at extreme ages of reproductive life in low- and middle-income countries. Notably, we found that neonatal mortality was significantly higher in this group compared to neonatal mortality rates reported regardless of maternal ages. Male babies, low and high birth-weighted babies, those born to mothers with no or low education, delivered at home, singletons, babies born with a small preceding birth interval, and those without postnatal checkups faced elevated risks of neonatal mortality. Additionally, neonates born in countries with high fertility and low literacy rates were also vulnerable. These findings underscore the urgent need for targeted interventions tailored to mothers at extreme ages. Policymakers and healthcare providers should prioritize strategies that address specific risk factors prevalent in these vulnerable populations. By doing so, we can improve neonatal outcomes and ensure the survival of these newborns during the critical neonatal period.

## Introduction

Neonatal mortality rate is defined as the number of deaths during the first 28 completed days of life per 1000 live births (LBs), and it is a significant global public health concern^1^. Although there has been a noticeable improvement in the last few decades in the lowering of newborn mortality, it remains a major concern for most low- and middle-income countries^2^.

Globally, 8.2 million children under the age of 5 die each year, and more than 40% of these are neonatal deaths, occurring before 30 days of life. In 2019, 2.4 million children died in their first month of life worldwide, with approximately 98% of the neonatal deaths occurring in low- and middle-income countries^3,4^. In low- and middle-income countries, Sub-Saharan Africa (SSA) had the highest neonatal mortality rate (27 deaths per 1000 LBs), followed by Central and Southern Asia (24 deaths per 1000 LBs)^5,6^. The rates of mortality appear far below the Sustainable Development Goal (SDG) 3.2 target of reducing the neonatal mortality rate to 12 or less per 1000 live births by 2030^7,8^. Despite the substantial decrease in under five and infant mortality rates, the decline in neonatal mortality is steady in low- and middle-income countries^9^.

According to the previous studies^10–15^, maternal education, deliveries without a skilled care provider, complications during pregnancy, birth weight, household wealth status, and a lack of basic antenatal and postnatal care utilization were among the factors statistically associated with mortality in the neonatal period in low and middle-income countries.

Neonatal mortality remains a critical public health issue, constituting a significant proportion of under-5 mortality in various regions. Paradoxically, neonatal health has received less attention compared to child mortality rates^16^. Our study aims to address this gap by investigating neonatal mortality rates among neonates born to mothers at extreme ages of reproductive life in low and middle-income countries. Specifically, we focus on the first 28 days of life—the neonatal period—which is widely recognized as one of the riskiest phases in human existence^17^. By examining this age group, we contribute to the understanding of neonatal health outcomes and identify potential risk factors associated with maternal age extremes. Our research seeks to bridge the knowledge gap and illuminate neonatal mortality patterns and associated factors, particularly among mothers at extreme ages. This understanding is crucial for developing targeted interventions and informed policy strategies. Importantly, our study builds upon existing evidence that maternally extreme ages significantly impact neonatal survival in low- and middle-income countries^18–20^.

## Methods

### Data source, study setting and population

The present study involves a secondary analysis of demographic and health surveys conducted between 2015 and 2022 in low- and middle-income countries. The study included a total of 43 countries that had standard Demographic and Health Surveys (DHSs) during this specified time period. These countries represent a diverse range of contexts and populations. The list of countries included in the analysis comprises Afghanistan, Albania, Angola, Armenia, Bangladesh, Benin, Burkina Faso, Burundi, Cambodia, Cameroon, Colombia, Côte d’Ivoire, Ethiopia, Gabon, Gambia, Guinea, Haiti, India, Indonesia, Jordan, Kenya, Liberia, Madagascar, Malawi, Maldives, Mali, Mauritania, Myanmar, Nepal, Nigeria, Pakistan, Papua New Guinea, Philippines, Rwanda, Sierra Leone, South Africa, Tajikistan, Tanzania, Timor-Leste, Turkey, Uganda, Zambia, and Zimbabwe. I accessed the DHS datasets from the Monitoring and Evaluation to Assess and Use Results Demographic and Health Survey (MEASURE DHS) program, which are available at https://www.dhsprogram.com/data/available-datasets.cfm in the public domain.

The source population included all live births born within the five years preceding the 2015–2022 standard DHSs in 43 low- and middle-income countries.

The study population included all live births born within the five years preceding the 2015–2022 standard DHSs in selected enumeration areas (sampling clusters) in 43 low- and middle-income countries. A total sample of 151,685 live births were enrolled into the analysis of this study.

### Variables and data management

The outcome variable of the study was neonatal mortality, which was dichotomized as yes if a baby died and no if it lived within the neonatal period. Considering the hierarchical nature of the data (household and cluster/community level) and the setting of the study at the inter-country level, the explanatory variables of the study were grouped into three levels. The first level included individual or household variables. The second level included community-level variables, and the third level included country-level variables.

**Figure 1:**
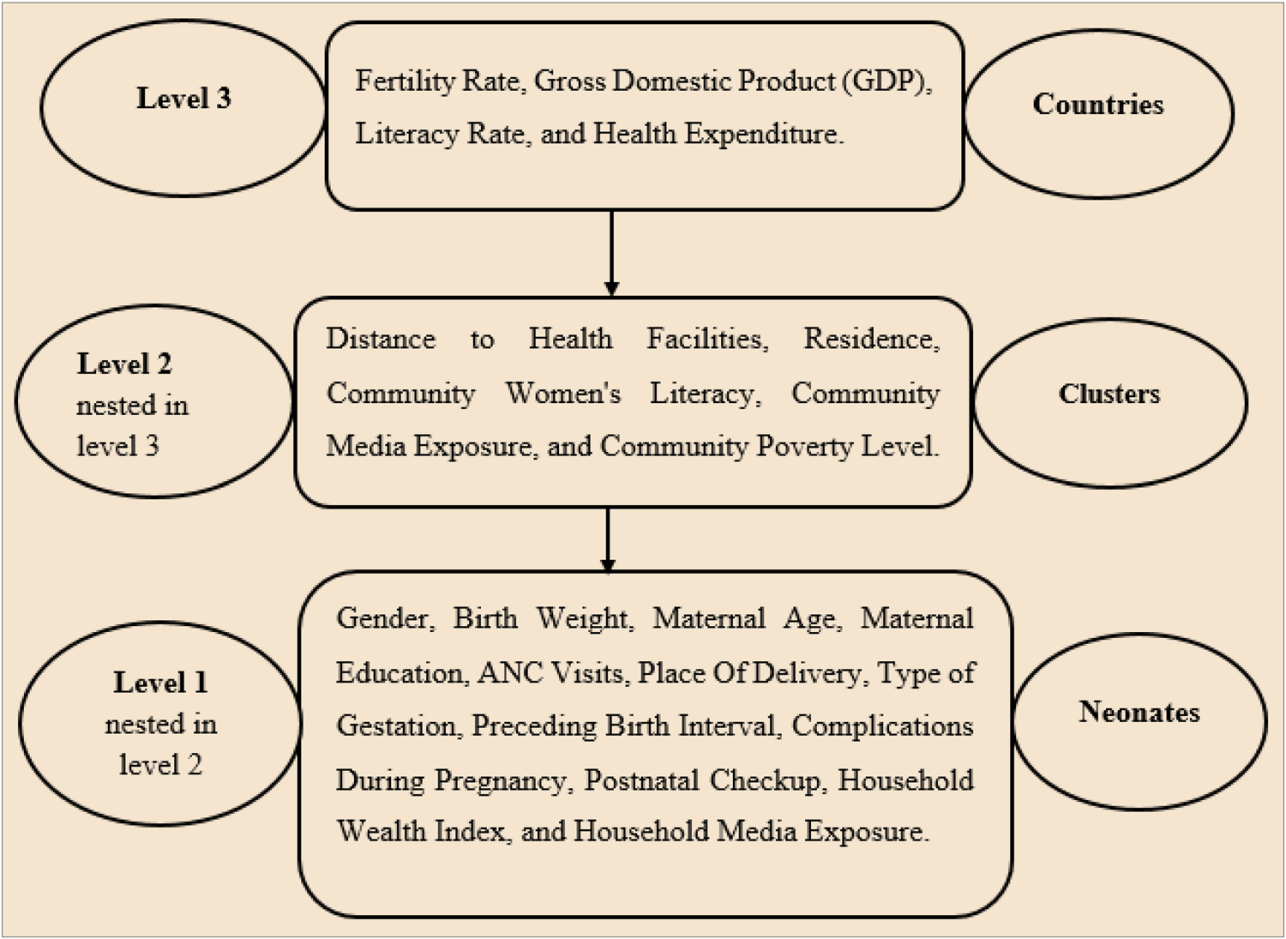
Level of variables included in the study.

At the first level, neonate gender, birth weight, maternal age, maternal education, ANC visits, place of delivery, type of gestation, preceding birth interval, complications during pregnancy, postnatal checkup, household wealth index, and household media exposure were included. At the second level, distance to health facilities, residence, community women's literacy, community media exposure, and community poverty level were included. At the third level, fertility rate, gross domestic product GDP in US dollars, literacy rate, and health expenditure are incorporated (Fig. 1).

In my analysis, I categorized community- and country-level variables into low and high groups. The median values served as the reference group for comparison. This approach not only accounts for nonlinear effects but also enhances interpretability in the policy context.

Maternal Age: Extreme ages of reproductive life encompass both adolescent mothers, typically ranging from 10 to 19 years old, and advanced maternal age, referring to mothers aged 35 years or older at the time of conception^21,22^. Notably, the Demographic and Health Survey (DHS) dataset we analyzed includes women aged 15–19 and 35–49, as standard DHS data collection focuses on women of reproductive age (15–49).

Control variable: Considering the different years of the DHS datasets used by the study, years of the DHS were used as a control variable to protect the differential time effect on the study findings.

The DHS datasets in Stata format were extracted separately from the MEASURE DHS program for all countries and appended together to create a single dataset. Stata 17 statistical software was used for data management and formal analysis. To draw the valid inference, weighting was done using weighting variables with consideration of the sampling design.

### The regression analysis

The three levels of variables employed in the study lead to a multilevel mixed effect model for regression analysis. Accordingly, five mixed effect models were applied by the study: the null model (a model without explanatory variables), model I (a model with first-level variables and the control variable), model II (a model with second-level variables and the control variable), model III (a model with third-level variables and the control variable), and model IV (a model with all three-level variables and the control variable). The three level regression model is equated as^23,24^:

$$ yijk = \beta 0k + \beta 1kxijk + rijk + uijk + eijk $$

where yijk is the dependent variable for the ith individual in the jth community in the kth country, β0k and β1k are the intercept and slope coefficients for the kth country, xijk is the independent variable for the ith individual in the jth community in the kth country, rijk is the random effect for the jth community in the kth country, uijk is the random effect for the ith individual in the jth community in the kth country and eijk is the residual error for the ith individual in the jth community in the kth country.

This study assessed both variation (random effect) and association (fixed effect). For the random effect, we evaluated parameters such as variance, intra-class correlation coefficient (ICC), and median odds ratio (MOR). The methodology for determining these parameters was described elsewhere^25^. As for the fixed effect, we used the adjusted odds ratio (AOR) within a 95% confidence interval (CI). An association was considered statistically significant when the p-value was less than the level of significance (0.05). Additionally, given the nested structure of the multilevel model, we employed log likelihood and deviance for model comparison. Multicollinearity was addressed using Stata and multivariable analysis. Specifically, Stata automatically handles multicollinearity by omitting variables with high correlation during the analysis.

### Ethical approval

This study was based on analysis of existing survey datasets in the public domain that are freely available online with all the identifier information anonymized, no ethical approval was required. The author obtained authorization for the download and usage of the archive of the DHS dataset of all countries included in the analysis from MEASURE DHS.

## Results

### Socio-demographic characteristics of study subjects

This comprehensive study included a total sample of 151,685 live births (comprising 76,994 males and 74,691 females) for analysis. Among the study subjects, 47.66% had normal birth weights. The majority of neonates (80.73%) were born to mothers aged 35–49 years, while 38.59% were born to mothers with no formal education.

Notably, over half (58.39%) of the study subjects were born after their mothers attended four or more antenatal care follow-ups, emphasizing the importance of prenatal healthcare. Equally significant, 70.10% of the births occurred at health facilities, highlighting the role of skilled attendance during delivery.

Regarding birth intervals, 84.24% of neonates had a preceding birth interval of 24 months or more. However, postnatal care remains an area of concern, as 61.59% of subjects did not receive a postnatal checkup.

Over one-fourth (26.19%) of the study subjects fell into the category of poorest wealth index, while a significant majority (73.75%) of households had limited or no exposure to media.

At the community level, 66.69% of study subjects were sampled from rural areas, and 69.78% were born in communities with low literacy rates. Additionally, 55.70% of subjects resided in communities characterized by low poverty levels.

Zooming out to the country level, 51.47% of study subjects were born in countries with low fertility rates. The majority (79.81%) of babies were born in countries with low literacy rates. Remarkably, 94.64% of the countries analyzed had low health expenditures (Table 1).

**Table 1:** Socio-demographic characteristics of the study subjects.

Variables	Response	Frequency (n)	Percent (%)
Individual level factors			
Gender	Male	76,994	50.76
	Female	74,691	49.24
Birth weight	Low	64,275	45.94
	Normal	66,692	47.66
	High	8953	6.40
Maternal age	15–19	29,234	19.27
	35–49	122,451	80.73
Maternal education	No education	58,532	38.59
	Primary	43,181	28.47
	Secondary	38,704	25.52
	Higher	11,269	7.43
ANC visits	< 4 visits	46,948	41.61
	≥ 4 visits	65,872	58.39
Place of delivery	Home	42,144	29.90
	Health facility	98,782	70.10
Type of gestation	Single	146,731	96.73
	Multiple	4,955	3.27
Birth interval	< 24 months	19,004	15.76
	24 or more months	101,561	84.24
Complications during pregnancy	Yes	25,581	71.09
	No	10,401	28.91
Postnatal checkup	Yes	43,225	38.41
	No	69,317	61.59
Household wealth index	Poorest	39,728	26.19
	Poorer	32,363	21.34
	Middle	29,146	19.21
	Richer	26,271	17.32
	Richest	24,178	15.94
Household media exposure	Yes	38,896	26.25
	No	109,252	73.75
Community level variables			
Distance to health facility	Big problem	54,793	37.81
	Not big problem	90,129	62.19
Residence	Urban	50,522	33.31
	Rural	101,163	66.69
Community women literacy	Low	105,842	69.78
	High	45,844	30.22
Community media exposure	Low	89,042	59.05
	High	61,744	40.95
Community poverty	Low	84,491	55.70
	High	67,195	44.30
Country level variables			
Fertility rate	Low	78,075	51.47
	High	73,611	48.53
GDP (US$)	Low	59,523	39.96
	High	89,430	60.04
Literacy rate	Low	121,057	79.81
	High	30,629	20.19
Health expenditure	Low	143,558	94.64
	High	8128	5.36

### Prevalence of neonatal mortality

**Figure 2:**
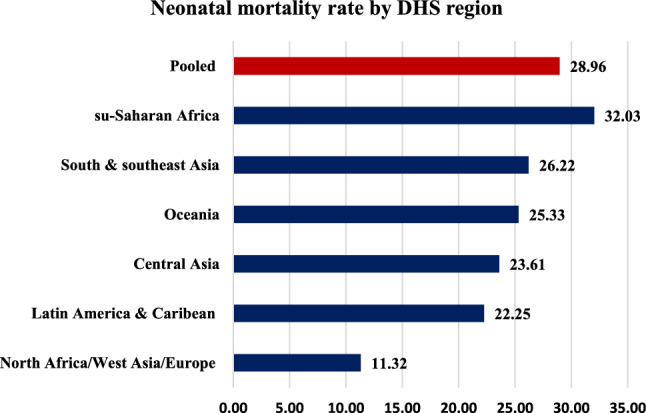
Prevalence of neonatal mortality rate by DHS region, 2015–2022 standard DHSs.

The pooled prevalence of neonatal mortality rates among neonates born to mothers at extreme ages of reproductive life in low and middle-income countries was found to be 28.96 neonatal deaths per 1000 live births at a 95% CI (28.13, 29.82). The high rate of neonatal mortality (32.03 neonatal deaths per 1000 live births) was observed in the sub-Saharan region of the DHS program (Fig. 2).

### Random effect (measures of variation) and model comparison

The values of variance in the null model imply that there was a significant variation in the neonatal mortality rate across the countries (τ = 0.578, p < 0.001) and across the communities (τ = 0.687, p < 0.001). The variation remains significant even after being controlled for all explanatory variables in the final model (model IV). According to the value of ICC for countries and communities in the null model, 12.7% and 27.8% of total variation in neonatal mortality rates were attributed to variation across countries and across communities, respectively. The unexplained heterogeneity (MOR) in neonatal mortality across countries and across communities was 2.06 and 2.20, respectively, and it was reduced to 1.64 and 1.66 after all levels of factors were fitted to the model. Model IV, a model with a large value of LLR (-2253.56) and a low value of deviance (4,507.12), was selected as the best-fit model for the measure of association (Table 2).

**Table 2:** The measures of variation (random effect) and model comparison.

Parameters		Null model	Model I	Model II	Model III	Model IV
Random effect						
Variance (τ)	Country	0.578**	0.336	0.475	0.483	0.242
	Community	0.687**	0.399	0.685	0.678	0.287
ICC (%)	Country	12.7%	4.9%	10.7%	17.5%	3.9%
	Community	27.8%	10.8%	26.1%	31.75%	8.0%
MOR	Country	2.06	1.73	1.92	1.93	1.64
	Community	2.20	1.82	2.19	2.18	1.66
Model comparison						
LL		− 202,265.17	− 2302.85	− 201,561.43	− 202,055.32	− 2253.56
Deviance		404,530.34	4,605.70	403,122.86	404,110.64	4,507.12

*ICC* intra-class correlation coefficient, *MOR* median odds ratio, *LL* log likelihood.

*P value < 0.001.

### The fixed effect (measures of association)

The multilevel mixed effect analysis of this study found that gender, birth weight, maternal education, place of delivery, type of gestation, birth interval, postnatal checkup, fertility rate, and literacy rate were significantly associated with the neonatal mortality rate.

The odds of neonatal mortality were 1.27 times higher among male neonates (AOR = 1.27 at 95% CI 1.06, 1.52) compared to females. Taking neonates with normal birth weight as reference, the odds of neonatal mortality were 5.49 times higher among neonates with low birth weight (AOR = 5.49 at 95% CI 4.48, 7.01) and 2.43 times higher among neonates with high birth weight (AOR = 2.43 at 95% CI 1.58, 3.74). Compared to babies born to mothers with higher educational attainment, the odds are 2.23, 1.93, and 1.85 times higher among babies born to mothers with no formal education (AOR = 2.23 at 95% CI 1.33, 3.74), primary schooling (AOR = 1.93 at 95% CI 1.15, 3.24), and secondary schooling (AOR = 1.85 at 95% CI 1.16, 2.96), respectively.

When compared to babies born at a health facility, babies born at home had 1.66 times higher odds of neonatal mortality (AOR = 1.66 at 955 CI 1.31, 2.10). Multiple (twins or triplets) gestational births had 5.65 times higher odds of neonatal mortality (AOR = 5.65 at 95% CI 3.90, 8.18), compared to singleton births. The odds of neonatal mortality were 1.50 (AOR = 1.50 at 95% CI 1.19, 1.90) times higher among babies with a birth interval of less than 24 months compared to babies born with a birth interval of 2 or more months. The odds of neonatal mortality were 3.50 times higher among babies who had no postnatal checkup (AOR = 3.50 at 95% CI 2.77, 4.44) compared to the reference group.

The odds of neonatal mortality were 1.25 times higher among neonates born in countries with high fertility rates (AOR = 1.25 at 95% CI 1.02, 1.56) compared to babies born in countries with low fertility rates. Moreover, the odds of neonatal mortality were 1.59 times higher among babies born in countries with a low literacy rate (AOR = 1.59 at 95% CI 1.06, 2.40) compared to babies born in countries with a high literacy rate. (Table 3).

**Table 3:** The measures of association (fixed effect) analysis.

Factors		Model I AOR (95% CI)	Model II AOR (95% CI)	Model III AOR (95% CI)	Model IV AOR (95% CI)
Survey year	2015–2019	1	1	1	1
	2020–2022	1.54 (1.26, 1.88)	0.87 (0.78, 0.97)	1.03 (0.91, 1.16)	1.19 (0.96, 1.49)
Gender	Male	1.27 (1.06, 1.53)			1.27 (1.06, 1.52)*
	Female	1			1
Birth weight	Low	4.79 (3.84, 5.96)			5.49 (4.48, 7.01)*
	Normal	1			1
	High	2.33 (1.53, 3.56)			2.43 (1.58, 3.74)*
Maternal age	15–19	1			1
	35–49	1.00 (0.60, 1.67)			0.81 (0.48, 1.37)
Maternal education	No education	1.72 (1.06, 2.80)			2.23 (1.33, 3.74)*
	Primary	1.81 (1.10, 2.98)			1.93 (1.15, 3.24)*
	Secondary	1.81 (1.14, 2.86)			1.85 (1.16, 2.96)*
	Higher	1			1
ANC visits	< 4 visits	0.97 (0.80, 1.18)			1.01 (0.83, 1.24)
	≥ 4 visits	1			1
Place of delivery	Home	1.65 (1.31, 2.08)			1.66 (1.31, 2.10)*
	Health facility	1			1
Type of gestation	Single	1			1
	Multiple	6.13 (4.26, 8.83)			5.65 (3.90, 8.18)*
Birth interval	< 24 months	1.52 (1.20, 1.91)			1.50 (1.19, 1.90)*
	24 or more months	1			1
Complications during pregnancy	Yes	1.10 (0.90, 1.34)			1.12 (0.91, 1.36)
	No	1			1
Postnatal checkup	Yes	1			1
	No	3.45 (2.73, 4.35)			3.50 (2.77, 4.44)*
Household wealth index	Poorest	1.26 (0.89, 1.77)			0.83 (0.56, 1.25)
	Poorer	1.22 (0.86, 1.73)			0.92 (0.63, 1.37)
	Middle	1.20 (0.84, 1.71)			1.02 (0.70, 1.48)
	Richer	1.36 (0.96, 1.91)			1.25(0.88, 1.78)
	Richest	1			1
Household media exposure	Yes	1.26 (0.97, 1.61)			1.28 (0.99, 1.63)
	No				1
Distance to health facility	Big problem		1.01 (0.94, 1.07)		1.03 (0.84, 1.26)
	Not big problem		1		1
Residence	Urban		1		1
	Rural		1.08 (1.01, 1.16)		1.05 (0.81, 1.35)
Community women literacy	Low		1.10 (1.01, 1.19)		0.90 (0.71, 1.15)
	High		1		1
Community media exposure	Low		1.08 (1.00, 1.17)		1.09 (0.87, 1.36)
	High		1		1
Community poverty	Low		1		1
	High		1.00 (0.93, 1.07)		1.16 (0.92, 1.46)
Fertility rate	Low			1	1
	High			1.27 (1.19, 1.35)	1.25 (1.02, 1.56)*
GDP (US$)	Low			1	1
	High			0.69 (0.58, 0.82)	1.42 (0.98, 2.06)
Literacy rate	Low			1.17 (1.01, 1.37)	1.59 (1.06, 2.40)*
	High			1	1
Health expenditure	Low			1.35 (1.12, 1.65)	0.73 (0.51, 1.05)
	High			1	1

*ANC* antenatal care, *AOR* adjusted odds ratio, *CI* confidence interval.

*Statistically significant (p-value < 0.05).

## Discussion

This study revealed the rate of neonatal mortality among neonates born to mothers at extreme ages of reproductive life in low and middle-income countries using data from recent standard demographic and health survey data taking the evidences show maternal extreme ages of reproductive life as a significant determinant neonatal mortality^18–20^ into account.

The pooled prevalence of neonatal mortality rate among neonates born to mothers at extreme ages of reproductive life in low- and middle-income countries was found to be 28.96 neonatal deaths per 1000 live births at a 95% CI (28.13, 29.82). The prevalence of neonatal mortality in this study was higher than the average global rate of 18 neonatal deaths per 1000 live births^26^. The rate of neonatal mortality in this study was also far higher than the global SDG target 3.2 of reducing the neonatal mortality rate (NMR) to 12 or fewer deaths per 1000 live births by 2030^27^. This implies that in low- and middle-income countries, neonatal mortality is high among neonates born to mothers at extreme ages of reproductive life. Therefore, neonates born to mothers at extreme ages of reproductive life in low- and middle-income countries need special emphasis to improve neonatal survival and/or reduce the neonatal mortality rate.

According to this study, the gender of the neonate, birth weight, maternal education, place of delivery, type of gestation, birth interval, postnatal checkup, fertility rate, and literacy rate were significantly associated with the neonatal mortality rate.

When compared to females, the odds of neonatal mortality rate were high among male babies. This finding agrees with findings from previous studies^28–30^. As per several studies on gender variation in fetal and perinatal outcomes, intra-uterine growth restriction, prematurity, respiratory distress syndrome, and birth asphyxia are more common among male babies than females^29,31–33^. Their larger heads and bodies also put them at a higher risk of mortality and birth injuries^29^. In addition, a meta-analysis of more than 30 million births also evidenced that stillbirths are tenfold higher among male babies as compared to females^34^.

The odds of neonatal mortality were high among low- and high-birth-weight babies as compared to normal-weight babies. This was in line with previous findings^35–37^. This could be due to the fact that low-birth-weight babies are often premature and have restricted fetal growth, while high-birth-weight babies are often born to mothers with diabetes or obesity, with all these problems leading a neonate to death^35^. Furthermore, low birth weight can result in problems for the infant, like hypoxia, poor physical growth, and respiratory and metabolic dysfunction, all of which raise the risk of infectious disease and malnourishment^38,39^.

Consistent with previous studies^11,40,41^, the odds of neonatal mortality were high among babies born to mothers with secondary school and lower educational status compared to babies born to mothers with higher educational attainment. This association can be attributed to several factors. Firstly, mothers with higher educational attainment often possess better health knowledge and awareness, allowing them to make informed decisions regarding prenatal care, nutrition, and recognizing potential health risks during pregnancy and childbirth^40,42^. Secondly, higher-educated mothers typically have better access to healthcare services and resources, including quality antenatal care and skilled birth attendants, which can lead to timely interventions and improved neonatal outcomes^43^. Additionally, mother's education is often correlated with socioeconomic status, with higher-educated women having better employment opportunities and income levels^40,42^. This improved socioeconomic status provides financial stability, enabling mothers to afford healthcare expenses, nutritious food, and a safe living environment, all of which contribute to better neonatal health outcomes. Furthermore, education empowers women, enhancing their decision-making abilities and enabling them to advocate for their own health and the health of their newborns^43,44^. Lastly, the association between mother's education and neonatal mortality may be mediated by attitudes towards modern health services and rejection of domestic violence^40^. Higher-educated mothers are more likely to have positive attitudes towards seeking healthcare services and rejecting domestic violence, which positively impact neonatal survival rates^44^.

This study also found that the home delivery was significantly associated with high odds of neonatal mortality. A systematic review and meta-analysis of 19 studies found that professional delivery at a health facility was associated with a 29% reduction in neonatal mortality in low- and middle-income countries^45^. The study also argues that it is imperative to create supportive environments, promote the use of medical facilities during childbirth, and expand facility delivery in areas where home delivery is common^45^.

The type of gestation was significantly associated with neonatal mortality. The odds of neonatal mortality were high among neonates born multiple compared to neonates born to singleton. This was in agreement with previous findings^46,47^. This may be related to the fact that babies of multiple gestational births are more likely to be premature, have low birth weights, have congenital defects, and are born to women who are older than average, all of which raise the risk of neonatal death^46,48^.

Consistent with previous findings, the neonates with shorter preceding birth intervals had increased odds of neonatal mortality compared to neonates with long birth interval^49–51^. This could be explained by the fact that short birth intervals lead to maternal depletion syndrome, a condition characterized by a mother's body being low in essential nutrients and minerals, which in turn could lead to poor fetal growth and development, which increases the risk of neonatal mortality^52^.

At the country level, this study found that the odds of neonatal mortality were high among neonates born in countries with high fertility rates compared to neonates born in countries with low fertility rates. This is in agreement with previous findings^53,54^. This could be due to the fact that low maternal health is frequently linked to high fertility rates, which may result in complications during pregnancy and childbirth^55^. High fertility rates can also result in poor living conditions and overcrowding, which raises the risk of newborn infections and other health issues^55^.

Furthermore, the odds of neonatal mortality were high among neonates in countries with a low literacy rate compared to neonates with high literacy rates. Similarly, it was affirmed that there is a positive correlation between the literacy rate and the neonatal mortality rate^56^. A higher literacy rate is associated with a lower neonatal mortality rate^56^. This is because a higher literacy rate is linked to better maternal health, which in turn leads to better neonatal health^56^.

When interpreting the findings of this study, it is important to consider the following strengths and limitations. The study demonstrates notable strengths, starting with the utilization of nationally representative large sample DHS (Demographic and Health Survey) data, which provides a significant advantage. By utilizing a large dataset, the study findings are expected to offer a more accurate representation of the neonatal mortality rate and its determinants in low and middle-income countries. This enhances the generalizability and reliability of the study's findings. Additionally, the study adopts a comprehensive approach by considering individual/household, community, and country-level factors as potential determinants of neonatal mortality. This multi-level analysis is a valuable strength as it allows for a more nuanced understanding of the complex factors influencing neonatal mortality rates. By incorporating various levels of factors, the study provides a comprehensive and holistic perspective on the determinants of neonatal mortality.

However, the manuscript also has a couple of limitations that should be acknowledged. Firstly, the study lacks data on clinical factors that may be associated with neonatal deaths. The absence of clinical data in the DHS dataset restricts the study's ability to explore the impact of medical factors on neonatal mortality rates. Future research endeavors should consider incorporating clinical data to facilitate a more comprehensive analysis. Secondly, the study does not directly compare specific factors between the younger and older extreme-age mothers groups. While the study examines the neonatal mortality rate and determinants among births of mothers at both younger and older extreme ages of reproductive life, it does not specifically analyze differences in subjective age between these two groups. Such a comparison would have offered valuable insights into how age-related factors influence neonatal mortality and could have enhanced our understanding of the unique challenges faced by each group. Addressing these gaps through further research would contribute to a more comprehensive understanding of neonatal health outcomes in diverse maternal age groups.

## Conclusion

This study sheds light on the neonatal mortality rates among neonates born to mothers at extreme ages of reproductive life in low- and middle-income countries. Notably, we found that neonatal mortality was significantly higher in this group compared to neonatal mortality rates reported regardless of maternal ages. Male babies, low and high birth-weighted babies, those born to mothers with no or low education, delivered at home, singletons, babies born with a small preceding birth interval, and those without postnatal checkups faced elevated risks of neonatal mortality. Additionally, neonates born in countries with high fertility and low literacy rates were also vulnerable. These findings underscore the urgent need for targeted interventions tailored to mothers at extreme ages. Policymakers and healthcare providers should prioritize strategies that address specific risk factors prevalent in these vulnerable populations. By doing so, we can improve neonatal outcomes and ensure the survival of these newborns during the critical neonatal period.

## Data Availability

The datasets generated and/or analyzed during the current study are available publicly online at MEASURE DHS [https://www.dhsprogram.com/data/available-datasets.cfm].
